# WTAP affects intracranial aneurysm progression by regulating m6A methylation modification

**DOI:** 10.1515/med-2023-0818

**Published:** 2023-10-16

**Authors:** Xuesong Yuan, Qing Bao, Bin Lu, Chong Xiang, Xiaoshan Hou, Wenfeng Wei

**Affiliations:** Department of Neurosurgery, Wujin Hospital Affiliated to Jiangsu University, Changzhou 213002, China; Department of Neurosurgery, Wujin Hospital Affiliated to Jiangsu University, No. 2 Yongning North Road, Tianning District, Changzhou 213002, China

**Keywords:** intracranial aneurysms, circle of Willis, m6A methylation, WTAP, rBMVECs

## Abstract

Intracranial aneurysm (IA) is a type of cerebrovascular disease that mainly occurs in the circle of Willis. Abnormalities in RNA methylation at the N6-methyladenosine (m6A) site have been associated with numerous types of human diseases. WTAP recruits the m6A methyltransferase complexes to the mRNA targets, and its expression is positively correlated with m6A methylation levels. This research aimed to explore the potential mechanisms of m6A methylation in IA. A selective arterial ligation method was used to establish an IA rat model; thereafter, the m6A methylation level and m6A methylation-related genes were determined in blood and circle of Willis samples using a commercial kit and real-time quantitative PCR, respectively. Subsequently, rat brain microvascular endothelial cells (rBMVECs) were treated with TNF-α, and the expression of m6A methylation-related genes within the cells were assessed. Lastly, the effects of WTAP on TNF-α-induced rBMVECs were further investigated through *in vitro* experiments. In result, the m6A RNA methylation level evidently declined in the blood and circle of Willis’ samples of the IA rats, as compared to the corresponding samples from the control rats (*P* < 0.05). Compared to the results in the control rats/cells, *WTAP* expression was significantly downregulated, whereas *ALKBH1* expression was evidently upregulated in the blood and circle of Willis samples of the TNF-α-induced rBMVECs of IA rats. Consequently, TNF-α-induced rBMVECs and rBMVECs with *WTAP* overexpression were successfully established. TNF-α inhibited the viability of the rBMVECs, promoted apoptosis, and significantly upregulated cleaved-caspase3 and downregulated WTAP expression. In contrast, *WTAP* overexpression significantly reversed these changes caused by TNF-α (*P* < 0.05). In conclusion, *WTAP* overexpression may modulate the growth of TNF-α-induced rBMVECs by enhancing *WTAP* expression and its m6A methylation.

## 1 Introduction

Intracranial aneurysm (IA) is a type of cerebrovascular disease characterized by the local dilation or expansion of blood vessels and is caused by weakness of the cerebral veins or cerebral artery walls [1,2]. It mainly occurs in the circle of Willis and affects about 3–5% of the total population [3]. The most frequent reason for a subarachnoid hemorrhage is the rupture of an IA; this is a devastating disease that can lead to death or permanent disability [4]. An unruptured IA is usually asymptomatic but can be recognized using magnetic resonance angiography as well as computed tomography angiography [5]. However, due to the high costs involved in brain imaging, IA screening for the entire population is not practical [2]. Despite evolvements in neurological intensive care, craniotomy, and endovascular coiling [6], meta-analysis studies have proved that the effects of the clinical treatments are not ideal, and the prognosis of IA is poor [7,8]. Therefore, it is imperative to further explore the underlying pathogenesis of IAs and unearth the novel potential biomarkers that will help diagnose and predict IA rupture in early stages; this will advance the survival as well as the prognosis of patients with the condition.

The gradual dilation and the eventual rupture of the intracranial artery are the natural processes of IAs. While the mechanisms of IA formation, growth, and eventual breakdown are not fully elucidated, it is known that the regulation of gene expressions (such as mRNA, rRNA, tRNA, long non-coding RNA, and microRNA) has important functions in IAs [3,9]. N6-methyladenosine (m6A) is the most abundant internal chemical modifier in RNA and is a crucial regulator of mRNA stability, protein expression, and multiple cellular processes, including cell differentiation and metabolism, splicing, stability and decay, processing, nuclear export, and translation [10,11,12]. Recent studies have indicated that m6A RNA methylation is strongly linked with the genesis and development of multiple types of cancers [13] as well as cardiovascular and cerebrovascular diseases [14]. Berulava et al. [15] demonstrated that the m6A structure was altered in cardiac hypertrophy and heart failure and that modifications in m6A RNA methylation can contribute to changes in protein levels; this indicated that m6A methylation may serve as a novel target for therapeutic intervention in heart failure. Another study demonstrated that in the endothelial cells under oscillatory stress, METTL3 was highly expressed; it was accompanied by a high level of m6A methylation, which implied that METTL3-mediated m6A methylation modification plays vital roles in the occurrence of atherosclerosis induced by oscillatory stress and the disturbance of blood flow [16]. Guan et al. reported that dysregulation of the m6A-related genes in gastric carcinoma (GC) patients was closely associated with the prognosis of the disease and that FTO could be a novel biomarker for GC prognosis [17]. All these reports have indicated that m6A methylation may offer more possibilities and new insights for the early diagnosis and treatment of various diseases. Additionally, lipopolysaccharide stimulation can enhance m6A methylation levels and trigger inflammatory cytokine production and pathways in liver tumors and dental pulps inflammation [18,19]. In rheumatoid arthritis, lipopolysaccharides can lead to increased levels of METTL3 expression as well as bioactivity in the macrophages; METTL3 overexpression can suppress the inflammatory response caused by the NF-κB pathway [20]. These reports demonstrated a strong association between m6A methylation modifications and inflammation [21]. However, the m6A methylation level and its related mechanisms in IA occurrence and development remain unclear.

Previous studies have confirmed that the inflammatory response is closely related to IA occurrence and advancement [22,23]. TNF-α is a crucial regulator of inflammatory responses and is highly released and accumulated in IAs [24]. In addition, a selective arterial ligation method has generally been used to construct IA models *in vivo*, which are histologically similar to humans [25,26]. Therefore, in this study, we aimed to investigate the potential mechanisms of m6A methylation and its related mechanisms in IA *in vitro* and *in vivo*. The *in vivo* and *in vitro* results showed that WTAP was significantly downregulated in the IA rats and TNF-α-induced rat brain microvascular endothelial cells (rBMVECs). WTAP, an important writer of m6A methylation, recruits the m6A methyltransferase complexes to the mRNA targets, and its expression is positively correlated with m6A methylation levels [27]. Zhu et al. reported that the WTAP expression in rat carotid arteries injured by balloon catheters was decreased; total Panax notoginseng saponin upregulated the decreased WTAP-p16 axis to inhibit intimal hyperplasia, which emphasized the roles of WTAP-p16 signaling in arterial restenosis [28]. Therefore, WTAP was selected to further investigate its roles in IA *in vitro*. This study will provide novel insights into the diagnosis and treatment of IAs and is expected to reveal novel potential therapeutic targets.

## 2 Materials and methods

### 2.1 Establishment of an IA rat model

Sixteen Sprague Dawley rats were purchased from Shanghai SLAC Laboratory Animal Co., Ltd (Shanghai, China). All rats were male and weighed between 200 and 250 g; they were fed in a controlled environment with a temperature of 24 ± 2°C, a humidity of 50 ± 5%, and a 12 h light/dark cycle. After acclimatization for 7 days, the animals were randomly divided into the control and IA groups (*n* = 8 for each group). Throughout the experiment, the animals were allowed to eat and drink freely.

In the IA group, rats were employed to construct a rat IA model using the selective arterial ligation method, as previously reported [26], with some modifications. Briefly, before modeling, the rats were made to fast for 8 h and were deprived of water for 2 h; then, they were generally anesthetized using a combination of sodium pentobarbital intraperitoneal injection (50 mg/kg) and isoflurane inhalation (2%). Following this, the rats underwent ligation of their left common carotid artery and electrocoagulation of the posterior branch of their ventral median renal artery. On the second day following surgery, 1% saline was given to the rats in the IA group instead of drinking water. The control group rats were not ligated and were given normal drinking water.


**Ethics approval and participation consent:** All the animal experiments were conducted in accordance with the relevant guidelines and regulations (National Medical Advisory Committee (NMAC) guidelines and ARRIVE guidelines Declaration of Helsinki) and were approved by the Institutional Animal Care and Use Committee of Changzhou Wujin People’s Hospital (approval no. [2022] 79). Additionally, the animal anesthesia procedures complied with the applicable veterinary guidelines (such as the American Veterinary Medical Association).

### 2.2 Sample collection and preparation

After 2 months of modeling, all rats were generally anesthetized with a combination of sodium pentobarbital intraperitoneal injection (50 mg/kg, China National Pharmaceutical Corporation) and isoflurane inhalation (2%), the process complied with the applicable veterinary guidelines (such as the American Veterinary Medical Association). Thereafter, the rats were intubated to the renal aorta through the left ventricle via a thoracotomy; blood samples were extracted using an EDTA tube and were preserved. Following this, 30 mL of normal saline was injected through a catheter, while the vena cava was cut open to drain the blood. Three rats were slowly injected through a catheter with 120 mL of 4% paraformaldehyde (China National Pharmaceutical Group Corporation)/0.1 M phosphoric acid buffer (pH 7.4, China National Pharmaceutical Group Corporation) for fixation; the other rats remained without this treatment. Thereafter, cervical dislocation was applied to kill the rats, and their brains were removed via craniotomy. The circle of Willis of each brain was carefully separated under a 10–16× surgical microscope. Among them, the three fixed circle of Willis samples were subjected to hematoxylin and eosin (HE) staining; the remaining five and blood samples were used to isolate the total RNA using the Trizol method.

### 2.3 Measurement of m6A methylation levels

The blood and circle of Willis samples in each group were employed for total RNA isolation using an RNAiso Plus kit (Trizol, Takara) in line with the manufacturer’s instructions. Thereafter, the isolated RNA was used for qualitative and quantitative detections, with a microplate reader (Thermo Scientific, Wilmington, DE, USA). Following this, the RNA m6A methylation levels in the blood and circle of Willis samples of each group were examined using an EpiQuik™ m6A RNA Methylation Quantification Kit (Colorimetric, Epigentek Group Inc., USA), according to the manufacturer’s protocols.

### 2.4 Quantitative reverse transcription PCR (RT-qPCR)

The aforementioned isolated RNA was reverse transcribed into cDNA using a commercial cDNA synthesis kit (Takara), according to the manufacturer’s instructions. The total RT-qPCR volume was 20 μL, and the displayed RT-qPCR reaction was as follows: 50°C for 3 min, 95°C for 3 min, a total of 40 cycles at 95°C for 10 s and 60°C for 30 s, followed by 95°C for 15 s, 60°C for 60 s, and 95°C for 15 s. The sequences of all the designed primers are shown in Table 1; *GAPDH* was a housekeeping gene. The relative mRNA levels of the related genes were obtained using the 2^−△△Ct^ method [29].

**Table 1:** Sequences of all the primers

Primer	Sequence (5′–3′)
METTL3	F: CTGGCACCCGAAAGATTGAG
	R: CATCTGGGTCCAGAAGGTGT
METTL14	F: GCAGAAACCTACGCGTCCTA
	R: CACCACGGTCAGACTTGGAT
WTAP	F: GCCTGGAAGTTTACGCCTGATA
	R: AATGGTGCTCTGCATACCCTCT
FTO	F: CCGTGGAACAAAGGAGTG
	R: GCAGAGGCATAGAAGGGT
ALKBH1	F: AGACAAGACTAAGCGGAGAC
	R: GAAAGCCAGGTCAGAAGG
VIRMA	F: CAGTCGTCGGGAAGGAACAA
	R: GACTAGGGCGGTAACCTGTG
TNFα	F: TCAGCCTCTTCTCATTCCTGC
	R: TTGGTGGTTTGCTACGACGTG
GAPDH	F: AGACAGCCGCATCTTCTTGT
	R: CTTGCCGTGGGTAGAGTCAT

### 2.5 Cell culture and cell transfection

The rBMVECs were acquired from Procell Life Science & Technology Co., Ltd (Wuhan, China) and were cultured in Dulbecco’s modification of Eagle’s medium (Gibco, USA), which contained 10% fetal calf serum (Gibco) and 1% penicillin/streptomycin (Gibco). The cells were stored in an incubator that was supplemented with 5% CO_2_ at 37°C and passed when the cells grew to 80–90% confluence.

The pcDNA3.1(+)-WTAP plasmid (oe-WTAP) and pcDNA3.1(+) plasmid (oe-NC) were prepared and synthesized by Yanzai Biotechnology (Shanghai) Co. Ltd; the cell transfection methods were the same as those reported previously [30]. The rBMVECs in good condition were harvested and inoculated into a six-well plate (6 × 10^5^ cells/well). Following overnight incubation, the serum-free medium was replaced with the original medium, and either 2.5 μg of pcDNA3.1(+)-WTAP plasmids or pcDNA3.1(+) plasmids were transfected into the rBMVECs using Lipofectamine 200 (Thermo Fisher Scientific). After 6 h of transfection, the complete medium was added and cultured for another 48 h. The RNA and proteins were extracted from the differently treated cells, whereas the WTAP mRNA and protein expressions were assessed using RT-qPCR and western blotting, to estimate the cell transfection efficiency. The *WTAP* sequences are displayed in Table 1.

### 2.6 Cell viability and apoptosis assays

The cells in good condition were collected and inoculated into a 96-well plate (5 × 10^3^ cells/well). Following overnight culture, the rBMVECs were randomly divided into either a control, TNF-α, or TNF-α + oe-WTAP group. First, the rBMVECs in the TNF-α and TNF-α + oe-WTAP groups were induced by TNF-α (2 ng/mL) for 48 h, and thereafter, the rBMVECs in the TNF-α + oe-WTAP group were transfected with the pcDNA3.1(+)-WTAP plasmid. The cells in the control group remained without treatment. Following 24, 48, and 72 h of culture, 10 μL of the cell counting kit-8 (Beyotime Biotechnology) reagent was supplemented to each well, and the mixture was incubated for another 2 h. Finally, a microplate reader was applied to test the absorbance at 450 nm.

The rBMVECs from the different treatments were acquired to determine cell apoptosis, using an Annexin V-FITC apoptosis assay kit (Beyotime Biotechnology). Briefly, the cells were resuspended with 1× Binding Buffer (100 μL), and thereafter 5 μL of Annexin V-FITC with 5 μL of propidium iodide was added. Following 15 min of incubation in the dark, at 25°C, 400 μL of 1× Binding Buffer was added; thereafter a flow cytometer was employed to acquire the cells, and the total cell apoptosis rate (early apoptosis rate + late apoptosis rate) was calculated.

### 2.7 Western blot

The rBMVECs were lysed using a RIPA lysis buffer (Beyotime Biotechnology) for total protein extraction, and a BAC assay kit (Boster, Wuhan, China) was employed to determine their concentrations. Subsequently, the protein samples were segregated using 10% SDS-PAGE, transferred to PVDF membranes, and then sealed using 5% skim milk. Following 2 h of incubation, the primary antibodies, including the anti-WTAP antibody (1:5,000, Proteintech Group, Inc.), anti-cleaved caspase3 (1:1,000, Proteintech Group, Inc.), and anti-GAPDH antibody (1:1,000, Proteintech Group, Inc.), were added to the membranes. After being incubated further, at 4°C overnight, the secondary antibody (goat anti-rabbit/mouse IgG-HRP, 1:5,000, Jackson ImmunoResearch) was added. Thereafter, following incubation at 37°C for 2 h, the protein bands were visualized using an ECL assay kit (Beyotime Biotechnology), and Image-Pro Plus (version 6.0, Media Cybernetics Imaging Technologies Inc., USA) was employed to quantify these protein bands.

### 2.8 Statistical analysis

All the data were expressed as the mean ± standard deviation, and each experiment was done in triplicate. The statistical analyses were conducted using GraphPad Prism 5 (GraphPad Software, Inc.). For differential analysis between two groups, Student’s *t-*test was performed; a one-way ANOVA followed by Tukey’s *post hoc* test was conducted for the multiple group analyses. The threshold for the statistical difference was set as *P* < 0.05.

## 3 Results

### 3.1 Histopathological changes and the m6A methylation level in an IA rat model

**Figure 1:**
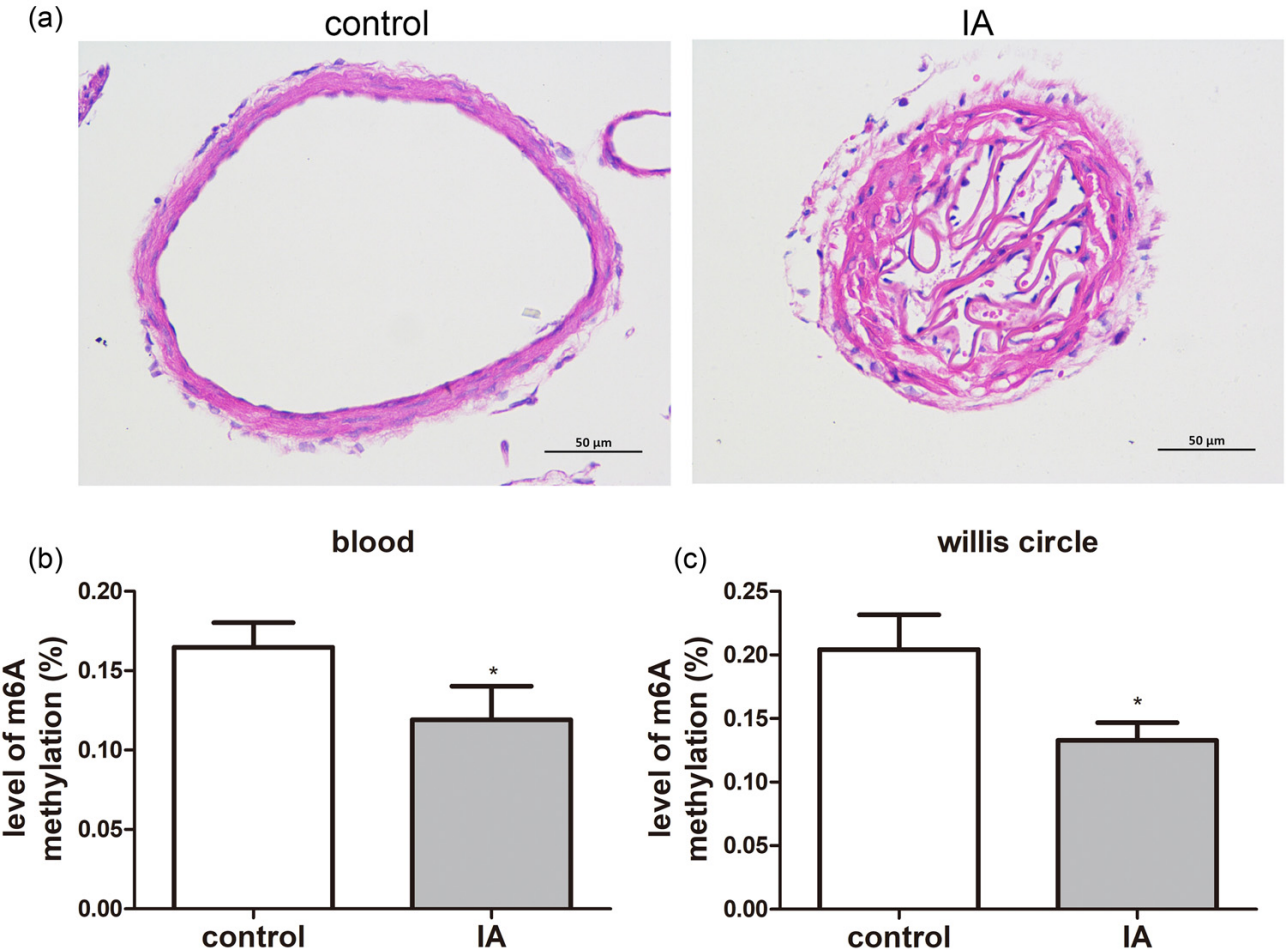
Histopathological changes of the circle of Willis and m6A methylation levels in an IA rat model. (a) Histopathological changes in the circle of Willis samples of the IA rats using HE staining. The m6A methylation levels in the blood samples (b) and circle of Willis samples (c) of the IA rats. *P < 0.05, as compared to the corresponding samples from control rats.

HE staining showed that the circle of Willis in the control rats were intact; whereas, in the IA rats, the inner elastic layer of the circle of Willis was damaged (Figure 1a). This indicated that the rat IA model was successfully established. Thereafter, the m6A methylation levels in the blood and circle of Willis samples of the control and IA rats were determined. It was clear that the m6A methylation levels in the blood and circle of Willis samples of the IA rats were both significantly decreased (*P* < 0.05) in comparison with the control rats (Figure 1b and c).

**Figure 2:**
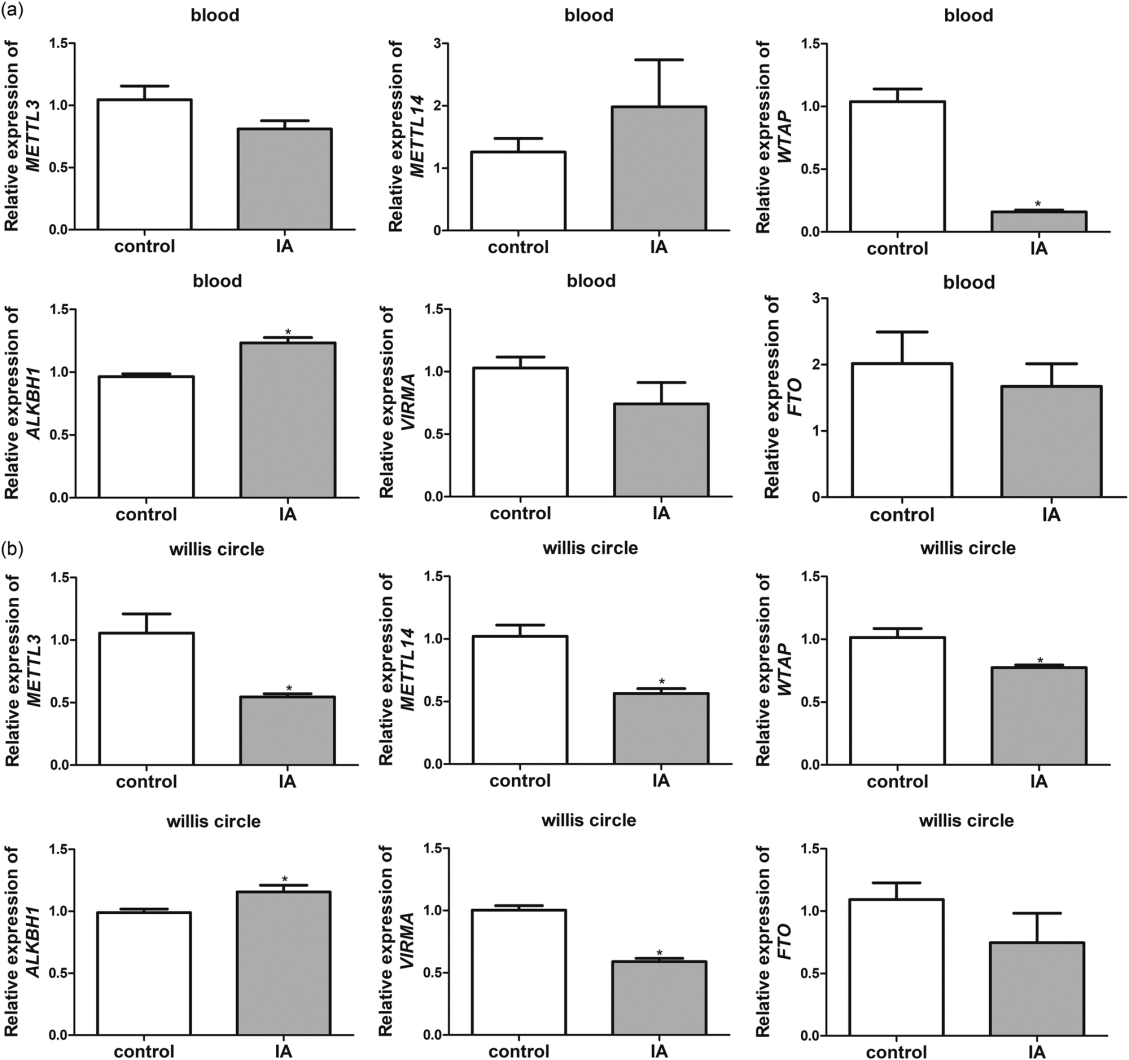
Relative mRNA expressions of the methylation-related genes (*METTL3*, *METTL14*, and *WTAP*) and demethylation-related genes (*ALKBH1*, *VIRMA*, and *FTO*) in the rats. Expressions of *VIRMA*, *METTL3*, *FTO*, *METTL14*, *ALKBH1*, and *WTAP* in the blood samples (a) or the circle of Willis samples (b) of the rats. *P < 0.05, as compared to the corresponding samples from control rats.

Thereafter, we assessed the expression level of the methylation-related genes (*METTL3*, *METTL14*, and *WTAP*) and demethylation-related genes (*ALKBH1*, *VIRMA*, and *FTO*) in the blood and circle of Willis samples of the different rats. In the blood samples, there were no significant differences (*P* > 0.05) in the relative expressions of *METTL3*, *METTL14*, *VIRMA*, and *FTO*, between the control and IA rats (Figure 2a). Relative to the control rats, *WTAP* expression was evidently downregulated (*P* < 0.05) whereas *ALKBH1* expression was markedly upregulated in the IA rats (*P* < 0.05, Figure 2a). Furthermore, in the circle of Willis samples, expressions of *METTL3*, *METTL14*, and *WTAP* were significantly downregulated in the IA rats, as compared to the samples from control rats (*P* < 0.05, Figure 2b). For the demethylation-related genes, it was observed that the *ALKBH1* expression was significantly elevated, whereas the *VIRMA* expression was evidently downregulated in the IA rats, as compared to the corresponding samples from the control rats (*P* < 0.05); the expression of *FTO* showed no obvious differences between the control and IA rats (*P* > 0.05, Figure 2b). All outcomes suggested that m6A methylation can be reduced in the IA rats.

### 3.2 Methylation-related gene expressions in the TNF-α-induced rBMVECs

**Figure 3:**
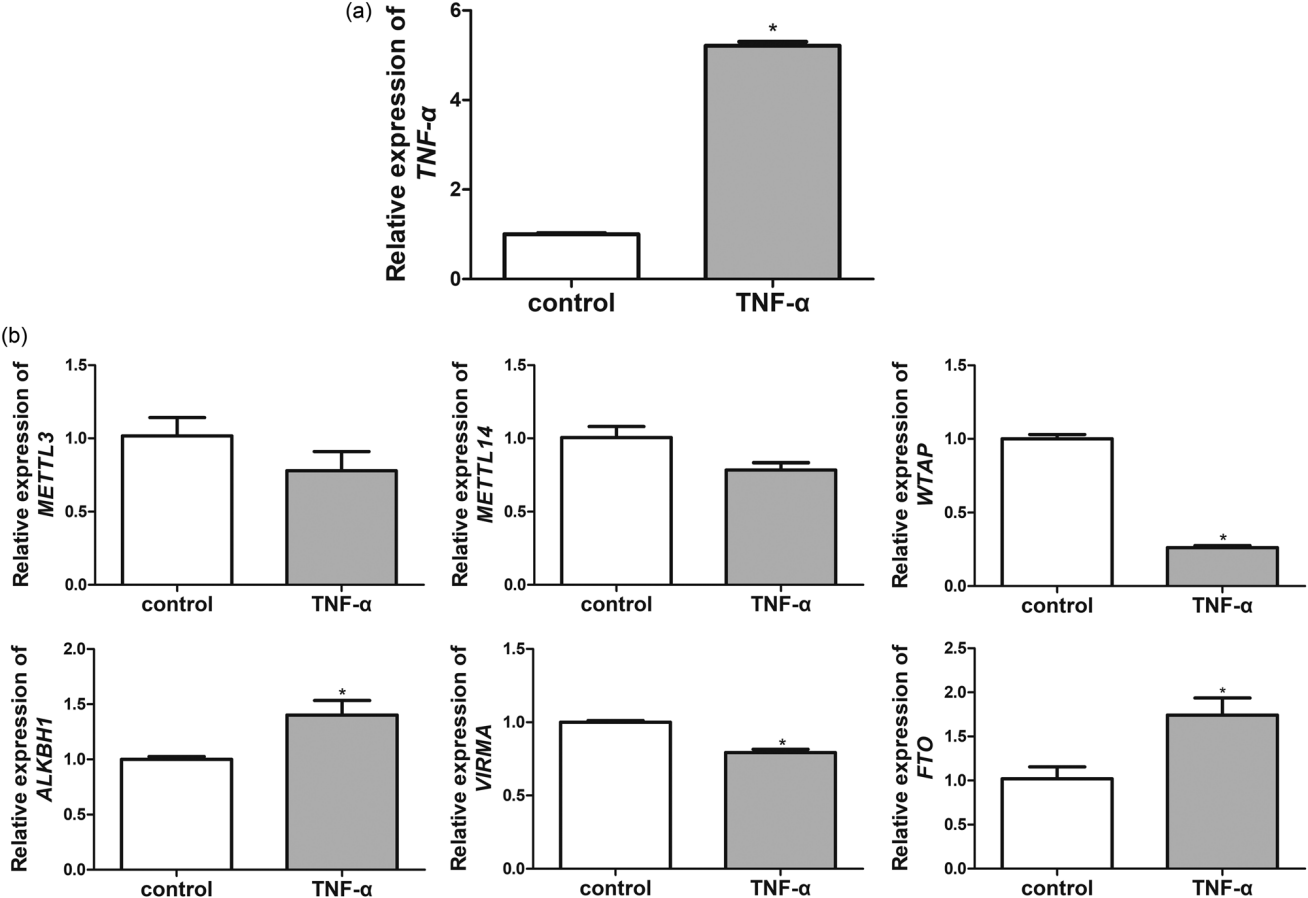
Expression of the methylation/demethylation-related genes in the TNF-α-induced rBMVECs. (a)Level of *TNF-α* in the cells treated with TNF-α. (b) Expressions of *ALKBH1*, *METTL3*, *VIRMA*, *METTL14*, *WTAP*, and *FTO* in the TNF-α-induced rBMVECs. *P < 0.05, as compared to the samples from the control cells.

TNF-α was applied to stimulate the rBMVECs in order to mimic the IA cell model *in vitro* [31]; thereafter, the *TNF-α* and methylation-related gene levels were determined. It was noted that the *TNF-α* mRNA expression in the control group and the TNF-α-induced rBMVECs were 1.00 ± 0.048 and 5.21 ± 0.155, respectively; this showed a significantly higher level of *TNF-α* in the TNF-α-induced rBMVECs than that of the control rBMVECs (Figure 3a). This indicated that the TNF-α-induced rBMVECs mimicked the IA model *in vitro* and could be used for further study.

Thereafter, it was found that TNF-α did not significantly influence *METTL3* and *METTL14* expression levels compared to those of the control rBMVECs (*P* > 0.05, Figure 3b). In comparison to the control rBMVECs, TNF-α induction evidently downregulated *WTAP* expression (*P* < 0.05) and upregulated *ALKBH1* expression (*P* < 0.05, Figure 3b). Moreover, *VIRMA* expression was significantly lower in the TNF-α-induced rBMVECs than the control rBMVECs (*P* < 0.05) but *FTO* expression was markedly upregulated in the TNF-α-induced rBMVECs (*P* < 0.05, Figure 3b). Due to the consistency and significance of RT-qPCR results in the blood, circle of Willis, and TNF-α-induced rBMVECs samples, *WTAP* was selected as the focus of our subsequent experiments.

### 3.3 Cell transfection efficiency

**Figure 4:**
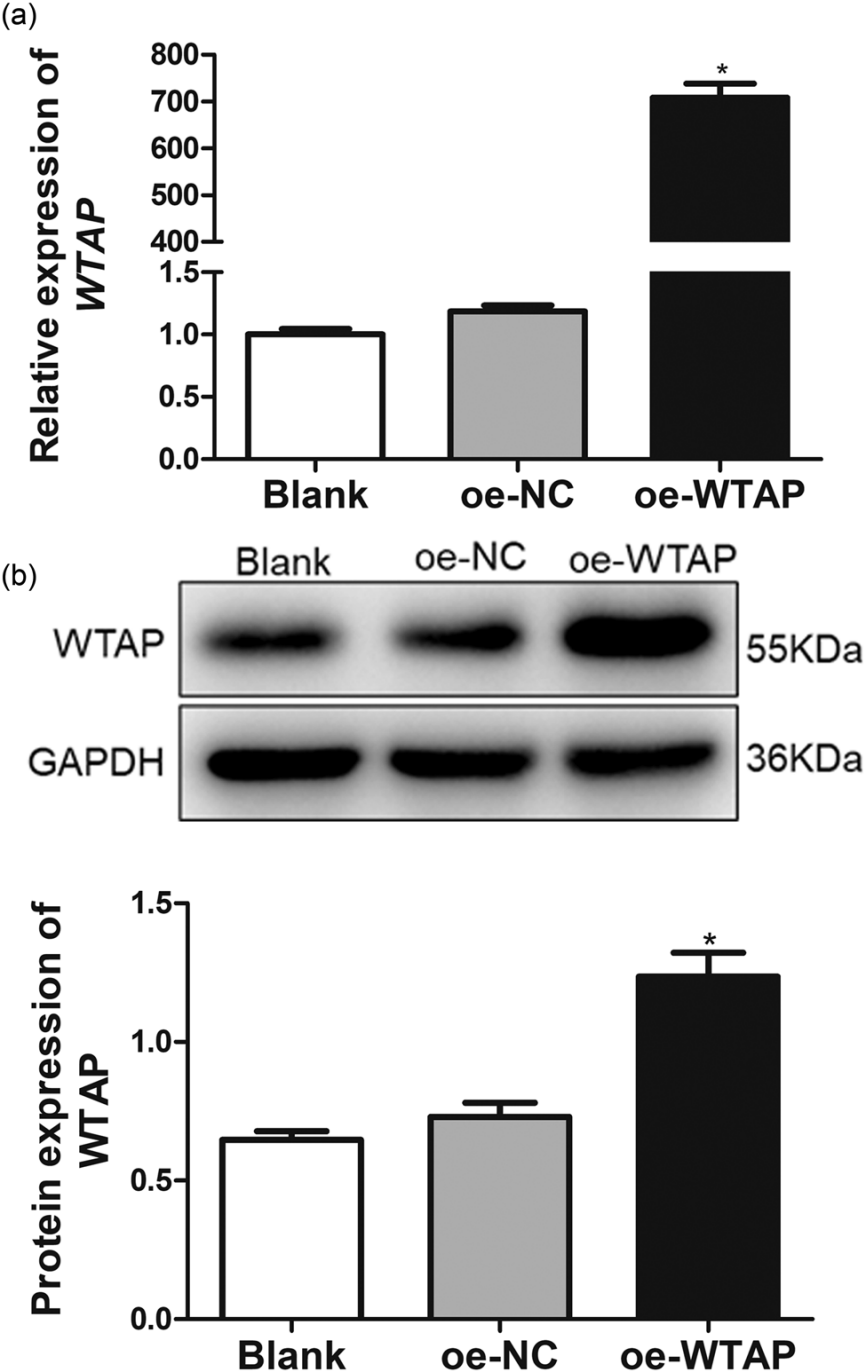
Cell transfection efficiency in the transfected rBMVECs. RT-qPCR and western blotting were employed to measure the WTAP mRNA (a) and protein (b) expression levels in the transfected rBMVECs. *P < 0.05, as compared to those of the blank cells.

The cell transfection efficiency was assessed by measuring the mRNA and protein expressions of *WTAP*. It was clear that the *WTAP* relative mRNA expression in the blank and oe-NC groups were 1.00 ± 0.075 and 1.19 ± 0.085, respectively; no significant difference was observed between the two groups (*P* > 0.05, Figure 4a). Nevertheless, when the rBMVECs were transfected with the pcDNA3.1(+)-WTAP plasmid, the relative expression of *WTAP* reached 707.92 ± 52.634, which was 700 times the value observed in the control group and significantly elevated compared to that of the blank cells (*P* < 0.05, Figure 4a). Additionally, the western blot analysis also showed that the WTAP protein expression in the blank and oe-NC cells exhibited no significant differences (*P* > 0.05), whereas following the transfection of the pcDNA3.1(+)-WTAP plasmid, the WTAP protein expression was evidently increased (*P* < 0.05, Figure 4b). All the findings indicated that rBMVECs with WTAP overexpression were successfully constructed and could be used for further study.

### 3.4 Roles of WTAP overexpression in the growth of TNF-α-induced rBMVECs

**Figure 5:**
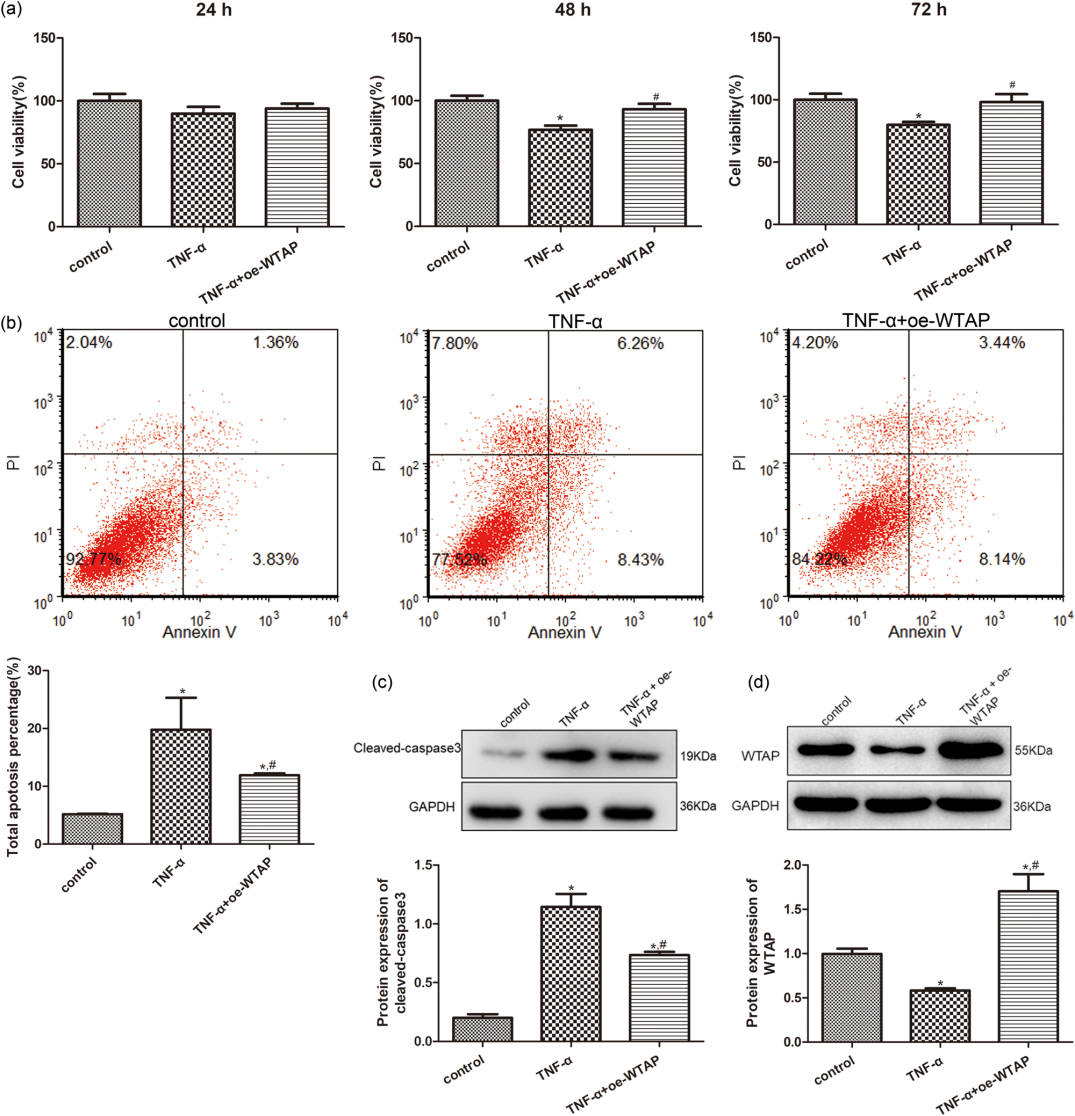
Roles of *WTAP* overexpression in the growth of the TNF-α-induced rBMVECs. (a) Cell viability of the rBMVECs, with different treatments, following 24, 48, and 72 h of culture. (b) Apoptosis of the rBMVECs, with different treatments, determined using flow cytometry. (c) Cleaved-caspase3 protein expression detected using western blotting. (d) WTAP protein expression detected using western blotting. *P < 0.05, as compared to the samples from the control cells; ^#^ *P* < 0.05, as compared to those from the TNF-α cells.

To further investigate the roles of WTAP on the growth of the TNF-α-induced rBMVECs, TNF-α was applied to treat rBMVECs. After 24 h of culture, the viability in the control cells, TNF-α-induced rBMVECs, and TNF-α-induced rBMVECs with *WTAP* overexpression showed no significant difference (*P* > 0.05, Figure 5a). However, after being incubated for 48 and 72 h the cell viability was significantly decreased (*P* < 0.05) in the TNF-α-induced rBMVECs compared to those in the control cells. Cell viability was significantly increased (*P* < 0.05) upon WTAP overexpression compared to that of TNF-α-induced cells, and recovered to a similar degree in control cells (*P* > 0.05, Figure 5a). Therefore, for cell apoptosis detection, the rBMVECs that were cultured for 48 h were selected.

Cell apoptosis of the rBMVECs induced by TNF-α was examined, and the acquired representative images of the different groups are shown in Figure 5b. The total cell apoptosis rates in the control, TNF-α, and TNF-α + oe-WTAP groups were 5.17 ± 0.082, 19.79 ± 5.52, and 11.92 ± 0.33%, respectively. These results indicated that TNF-α induction significantly increased (*P* < 0.05) the cell apoptosis of the rBMVECs compared to the results of the control cells. Cell apoptosis in the TNF-α + oe-WTAP rBMVECs was evidently reduced (*P* < 0.05, Figure 5b), compared to that in the rBMVECs induced by TNF-α. These results revealed that TNF-α can enhance the apoptosis of rBMVECs, whereas WTAP overexpression can inhibit the apoptosis caused by TNF-α.

Finally, the protein expressions of cleaved-caspase3 and WTAP in the cells with different treatments were detected. It was found that the tendency of cleaved-caspase3 protein expression in the different cells was similar to that of the cell apoptosis in the different groups (Figure 5c). WTAP protein expression was evidently downregulated (*P* < 0.05) in the TNF-α-induced rBMVECs compared to that in the control cells. However, relative to TNF-α-induced rBMVECs, WTAP protein expression was significantly upregulated (*P* < 0.05) in the TNF-α-induced rBMVECs with *WTAP* overexpression (Figure 5d).

## 4 Discussion

IA is characterized by the phenotypic regulation of vascular smooth muscle cells and the loss of vascular cells, posing a significant threat to human life and health [4]. It has been reported that m6A RNA methylation abnormalities are related to many types of human cancers, including brain tumors and breast and bladder cancers [32]. However, a few studies have reported the effects of m6A RNA methylation in IA. Our study showed, for the first time, the significantly low m6A RNA methylation levels in the blood and circle of Willis samples of IA rats. After measuring the related gene expressions, we found that *WTAP* expression was significantly downregulated, whereas *ALKBH1* expression was evidently upregulated in the blood and circle of Willis samples of the IA rats and TNF-α-induced rBMVECs. Additionally, we successfully established rBMVECs with *WTAP* overexpression, and observed that TNF-α inhibited the viability of rBMVECs, promoted apoptosis, and downregulated WTAP expression; *WTAP* overexpression significantly reversed these changes caused by TNF-α. These results will contribute to our comprehension of the important m6A methylation functions in IA progression.

Several previous studies have shown that m6A RNA modifications produce vital effects on both physiological and pathological conditions, especially in the evolvement and progression of different types of human cancers [13,32,33]. Methylation of m6A is reversibly and dynamically regulated by methyltransferase and demethylase, and the m6A binding proteins work by recognizing and binding to the m6A site of the target RNAs [14,34]. Our results showed that the m6A RNA methylation level was significantly reduced in the IA rats. To elucidate the specific molecular mechanisms, the expressions of *FTO*, *METTL3*, *ALKBH1*, *METTL14*, *VIRMA*, and *WTAP* were determined. Furthermore, WTAP, METTL14, and METTL3 are responsible for catalyzing m6A, whereas ALKBH1, VIRMA, and FTO serve as erasers to remove m6A [35,36]. Yue et al. [37] indicated that VIRMA was able to recruit and guide the core methyltransferase complex METTL3/METTL14/WTAP to specific RNA regions for m6A methylation. Another study showed that abnormal expressions of the demethylase genes *FTO* and *ALKBH1* had a significant prognostic value in GC patients, implying that these genes may have crucial functions in the development and metastasis of GC [38]. From these reports and our results, we concluded that the m6A methylation level can be decreased in IAs, accompanied by downregulated *WTAP* expression and upregulated *ALKBH1* expression.

Owing to the consistency and significance in the blood, circle of Willis, and TNF-α-induced rBMVECs samples, *WTAP* was selected for the subsequent experiments. A study of Jia et al. [39] demonstrated that miR-133a-3p enrichment can suppress the apoptosis of vascular endothelial cells and promote their proliferation and migration, thereby inhibiting IA progression. Another study also demonstrated that endothelial dysfunction was considered the first step in the pathogenesis of IAs; high levels of inflammation and apoptosis of the vascular endothelial cells can promote IA progression [40]. Therefore, rBMVECs were used in this study. In our study, rBMVECs with *WTAP* overexpression were successfully constructed, and the *WTAP* overexpression enhances the viability of TNF-α-induced rBMVECs, inhibits apoptosis, and significantly downregulates cleaved-caspase3, while upregulating WTAP expression. WTAP can interact with the METTL3–METTL14 complex [41] and is essential to initiate and guide nuclear spot localization, which is necessary for the activation of m6A methylation [27]. Additionally, WTAP has been identified as a nuclear protein that participates in the regulation of cell proliferation and apoptosis. Recently, WTAP was found to be overexpressed in glioblastoma; its ablation repressed the migration and invasion of the glioblastoma cells by regulating EGFR activity [42]. Another study illustrated that WTAP was highly expressed in hepatocellular carcinoma (HCC) and that WTAP-mediated m6A modifications could promote HCC progression via the HuR-ETS1-p21/p27 axis [43]. Another study reported that WTAP can facilitate osteosarcoma oncogenesis by inhibiting the expression of HMBOX1 in an m6A methylation-dependent manner [44]. In summary, it can be inferred that *WTAP* overexpression enhances the viability and suppresses the apoptosis of TNF-α-induced rBMVECs by down-regulating cleaved-caspase3, upregulating WTAP expression, and increasing its m6A methylation, thereby playing important roles in IA progression.

## 5 Conclusions

Lower m6A methylation levels were observed in the IA samples, and *WTAP* and *ALKBH1* may have potential as novel biomarkers for IAs. However, a large number of samples are required to confirm this finding. Additionally, *WTAP* overexpression may improve IAs by regulating the growth of rBMVECs and increasing their m6A methylation. Our findings help improve our understanding of the role of m6A methylation in IA progression, thereby laying the foundation for *WTAP* as a novel therapeutic target for IAs.
